# Examining the relationship between blood lead level and stunting, wasting and underweight- A cross-sectional study of children under 2 years-of-age in a Bangladeshi slum

**DOI:** 10.1371/journal.pone.0197856

**Published:** 2018-05-24

**Authors:** Mohammad Jyoti Raihan, Emily Briskin, Mustafa Mahfuz, M. Munirul Islam, Dinesh Mondal, Md Iqbal Hossain, A. M. Shamsir Ahmed, Rashidul Haque, Tahmeed Ahmed

**Affiliations:** 1 Nutrition and Clinical Services Division, International Centre for Diarrhoeal Disease Research, Bangladesh; Dhaka, Bangladesh; 2 Department of Epidemiology of Microbial Diseases, School of Public Health, Yale University, New Haven, Connecticut, United States of America; 3 School of Population Health, University of Queensland, Brisbane, Australia; BRAC, BANGLADESH

## Abstract

Elevated blood lead level (BLL) is known to cause cardiac, immune, and cognitive damage but had not been thoroughly studied in relation to stunting among children under two years of age. We primarily aimed to assess the relationship between elevated BLL, the accumulation of concerned amount of the metal lead in blood and stunting and secondarily—wasting and underweight amongst Bangladeshi children less than two years of age. For this cross-sectional study, BLL measurements, anthropometric data, and socioeconomic indicator information were collected and analyzed for 729 children under two years of age upon enrollment in the MAL-ED study conducted in a Bangladeshi slum area. Univariate, bivariate and multivariate analyses were carried out to observe the proportion and mean and contribution of elevated BLL and other relevant variables in explaining the occurrence of stunting. Of the enrolled subjects, 39.0% were stunted [length-for-age z score (LAZ<-2)], 50.3% were male, and 86.6% had an elevated BLL (≥5μg/dL). Mean BLL of stunted children was 8.47 ± 3·37 μg/dL and 8.10 ± 3·80 μg/dL for non-stunted children. Proportion of children with elevated BLL was not significantly different between the stunted and non-stunted groups (p>0.05). When adjusted for other variables, elevated BLL was found to be a significant predictor of stunting and underweight (p<0.05) but not wasting (p>0.05). Elevated BLL (p<0·01), child’s gender and weight (p<0·001), maternal body mass index (BMI) (p<0.05) and severe household food insecurity (p<0·05) were all significantly associated with stunting in the multivariate model. Increased odds of stunting was also observed for increased BLL. The findings suggest that chronic lead poisoning is significantly associated with high level of stunting among child slum dwellers in Bangladesh. These findings strengthen the argument for improved lead reduction efforts in Bangladesh, where lead poisoning and stunting are both highly prevalent.

## Introduction

Lead poisoning, or elevated BLL, which is the accumulation of the metal -lead at high level in blood [1], causes cardiovascular, immune and permanent cognitive damage and contributes to 0·6% of the global burden of disease [2]. Lead does not have any essential physiological role in human body [1] and chronic lead poisoning, a ‘*man made disease* [3], is caused by accumulation of a large dose of lead which in addition to many vital organs also affects the central nervous system leading to coma, convulsions, and even death. Lower degrees of BLL, as low as <5μg/dL, cause no obvious symptoms and were previously considered safe [4]. In 2004, 16% of all children worldwide were estimated to have BLL above the CDC suggested cut-off value of 10 μg/dL [2], however, more recent evidence suggests value of blood lead concentration above 5 μg/dL may cause adverse health effects, though it is now understood that there is no evidence suggesting a safe level of lead exposure in children [5, 6]. Current costs of childhood lead poisoning, including economic costs incurred due to neurobehavioural toxicity pertinent to lead poisoning, as well as indirect economic costs due to lost productivity worldwide are estimated to be US$43 billion per year [1]. While the relationship between elevated BLL and cognitive damage is well known, the association between malnutrition and lead poisoning requires further study.

Lead has been primarily classified as a neurotoxin [7, 8] and is found to be associated with developmental and behavioral problems in children [1, 9, 10]. Lead poisoning can be especially detrimental in pregnant women and young children living in low socio-economic condition, who are often more likely to ingest lead-contaminated dirt and dust [11]. Lead exposure is commonly associated with decreased immune function, especially in children [12, 13]. High maternal cumulative lead burden has been found to be associated with decreased genomic DNA methylation in cord blood, which could influence epigenetic programming, potentially increasing disease susceptibility and negatively affecting growth throughout the life course [14]. Nutritional inadequacies, such as irregular food intake, low calcium intake and subtle iron deficiency can increase the absorption of lead [15, 16] and they are common in slum environment.

Several studies have been found to address how undernutrition could increase lead absorption, but very few studies commented on how elevated BLL might cause stunting (height for age z-score or LAZ<-2), a profound and chronic form of undernutrition. A cross-sectional study in Bangladesh found that high BLL is associated with low BMI (p<0·001), indicating that compromised nutritional status increases the risk of lead poisoning. After adjusting for other variables/confounders, however, the proximity to industries and the use of indigenous medicines were found to be the only significant predictors of high BLL [17]. As poor families are more likely to live in lead-contaminated areas and to have undernourished children, socioeconomic status could be an important contributor when studying lead and undernutrition. Maternal anthropometric factors such as maternal height are also known to be associated with the child’s nutritional status, and must be accounted for whenever child stunting is studied [18, 19].

The Bangladesh Demographic and Health Survey (BDHS) of 2014 indicates that the current prevalence of stunting among children under five years of age is 36%, while the highest age group affected (46.3%) is the 18–23 months of age group [20]. The Government of Bangladesh has taken strong initiative to decrease the prevalence of undernutrition in the community as reflected by the operational plans under the five year Health, Population and Nutrition Sector Development Program (HPNSDP) which intends to significantly improve the nutrition status of women and children [21]. The move to improve children’s nutritional status in the country demands that the causes of child undernutrition be fully identified and studied so that they can be eliminated. Therefore, it is imperative to identify the gaps in knowledge about the risk factors for undernutrition in the community.

In light of the relationship between elevated BLL and stunting in children, several studies have shown a relationship between increased BLL and decreased height, but did not comment on the occurrence of stunting [22, 23]. Fewer studies have examined the relationship between elevated BLL and stunting in young children, and evidence suggests that increased BLL could lead to increased risk for stunting among children of all ages [24–27]. However, the age group considered for the studies are different. Therefore, to understand the role of elevated BLL in stunting among young children, the primary aim of this paper is to explore the relationship between BLL and stunting among children less than 2 years of age–the first 1,000 days of life, the critical period for child development [28]. Furthermore, this paper, as a secondary objective, is to investigate the relationship of elevated BLL with other manifestations of undernutrition such as wasting and underweight.

## Materials and methods

### Study design and sample size

**Fig 1:**
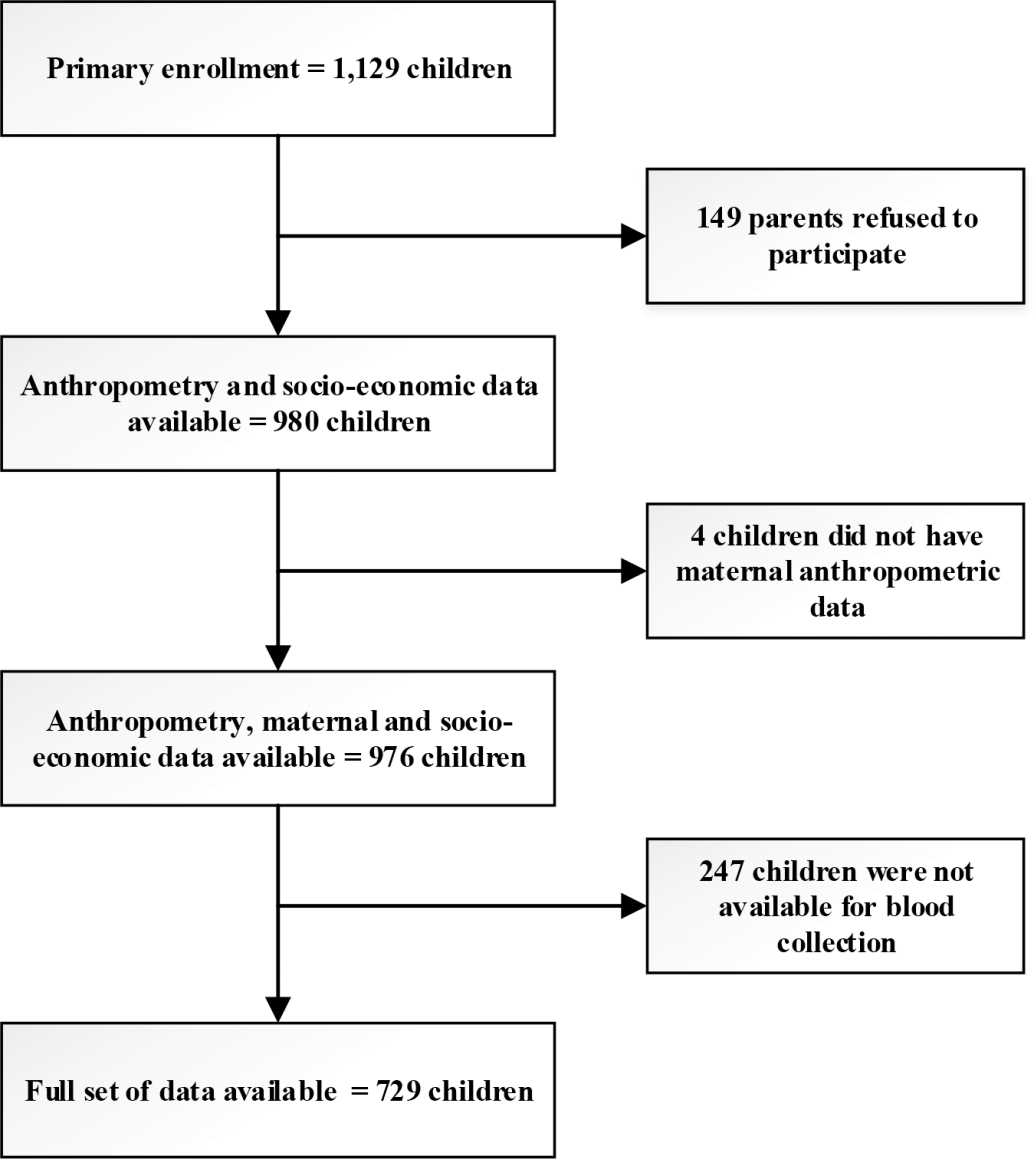
Trial profile.

The cross-sectional data of the 980 children under two years of age was collected through case-control component of The Interactions of Malnutrition & Enteric Infections: Consequences for Child Health and Development (MAL-ED) study in Bangladesh [29]. In total 1,129 children were primarily enrolled but 149 (13%) of the sample refused to participate. Anthropometry, maternal and socio-economic data were collected from all 976 participants. However, blood sample was available from 732 participants. In total full set of data was available from 729 participants. The MAL-ED study site in Bangladesh is located in urban Mirpur, one of the 21 administrative units of the capital city Dhaka, covering around 14.2 km^2^ and houses around half a million people mostly belonging to poor and middle class families. The particular MAL-ED site, where this study was undertaken, is situated in section 11 of Mirpur and consists the *Buniabadh* area, a typical urban slum dwelled by underprivileged families, with congested housing and suboptimal sanitation. The MAL-ED site is divided into five blocks, each containing around 1200 households. The data collection was carried out at enrolment by trained field workers between November 2009 and December 2012. The trial profile is shown in Fig 1.

### Ethical approval and consent procedure

This study was approved by the Internal Review Board (IRB) of icddr,b in Bangladesh, with separate approval from both the Research Review Committee (RRC) and Ethical Review Committee (ERC) of icddr,b. Informed written consent was taken from the parents or primary caregiver of the children. All consent givers were briefed and assured about non-disclosure of the information and use of the analyzed data findings for scientific purposes. However, in view of the chronic nature of the condition of elevated BLL, the researchers are screening the children for referral to a tertiary care hospital should there be abnormal symptoms and signs.

### Blood lead level measurement

BLL were measured using Graphite Furnace Atomic Absorption Spectroscopy (GAAS) [30, 31]. The M Series Atomic Absorption Spectrophotometer (M5 and MQZ) from Thermo Electron Corporation UK fitted with GF95 Zeeman Graphite Furnace with GFTV and FS95 Furnace Auto sampler was used for all analyses. Whole blood samples, QC, Pool and standards were diluted 10 times with modify reagent. Concentrations of unknown samples were determined against the standard curve. The detection limit for lead is 0.19 ppb. The accuracy and precision of the results were determined by NIST SRM 955c Caprine blood and pool samples. All tests were carried out at the Nutritional Biochemistry Laboratory of icddr,b (formerly known as International Centre for Diarrhoeal Disease Research, Bangladesh) in Dhaka, Bangladesh.

### Anthropometry and other variables of interest

Maternal and child nutrition status were measured through anthropometry. Child’s length and weight were measured using Seca 417 infantometer (precision: ± 1mm) and Seca 354 Dual Purpose Baby Scale (precision: 10 gm) respectively. After explaining to the mother about the purpose of the length and weight measurement, one trained field staff, the primary measurer, placed the incumbent child in a supine position on the infantometer with the crown of the head touching the headboard. Another field staff maintained the head position during the measurement. The legs were then extended at the hips and knees by the primary measurer and held flat on the board with one hand while the measurer slides the movable board against the heels with his/her other hand. The length was recorded to the nearest 0.1 cm. While, the child being nude or in a clean and dry diaper were usually laid down to be weighed. However, if the child was not calm being laid down, weight measurement was carried out with the child being seated on the weight scale. The child was played with or distracted while getting the measurement. The primary measurer maintained the safety of the child and read the weight aloud and the other field staff noted the weight. Weight was recorded to the nearest 10 g or 0.01 kg. Maternal height and weight were recorded using Seca 217 stadiometer (precision: ± 1mm) and Seca digital scale for adults (precision: 10 gm). While, for measuring height, the mother was requested to stand in an upright position against the board. Any issues regarding hats/scarfs, headdress, or hairstyle which could have made measurement difficult was resolved before the measurement. The primary measurer ensured that the subject’s heels were together if possible and touching the board and the shoulders and buttocks were toughing the board as well. The measurer also ensured that the mother was standing up straight while keeping her head up and staring forward. Maternal height was recorded to the nearest 0.1 cm. The mother was weighed in light clothing, the weight of which was estimated. The scale was placed on a flat surface, and the mother was requested to stand still on the scale until the scale displayed solid numerical values. The primary measurer read the weight aloud and the other field staff noted it. Maternal weight was recorded to the nearest 100 g or 0.1 kg. Relevant anthropometric measurements were converted to z-score using the *ZSCORE06* package of STATA [32] to represent appropriate anthropometric maternal and child variables [33].

A standard DHS questionnaire [34] was employed to collect other maternal and household information. Information of household food insecurity status was collected using Household Food Insecurity Access Scale (HFIAS), an experience-based scale which assesses the degree of food insecurity in the household for past 30 days [35]. The gradient of food security at the household level was categorized into food secure, mildly food insecure, moderately food insecure and severely food insecure. Maternal education status was dichotomized into ‘never attended school’ representing those mothers who never received any education from formal institution and ‘attended school’ constitutes the mothers who had at least some education from any formal institution. Information on household income was collected in Bangladeshi currency unit–Taka (Tk.). The participant’s selection process for MAL-ED study along with all instruments used for maternal and socioeconomic data is described elsewhere in details [32].

The cut-off values of different variables used during analysis are listed below in Table 1.

**Table 1:** Cut-off value definitions.

Lead group [36]	normal (BLL <5μg/dL)	elevated (≥5μg/dL)		
Stunting [37]	not stunted (LAZ ≥ -2)	stunted (LAZ<-2)		
Wasting [37]	not wasted (WLZ ≥ -2)	wasted (WLZ<-2)		
Underweight [37]	not underweight (WAZ ≥ -2)	underweight (WAZ<-2)		
Food insecurity score (HFIAS) [35]	food secure	mildly food insecure access	moderately food insecure access	severely food insecure access

### Statistical analysis

Exploratory analyses involved examination of the distribution of each variable and inter-relationships between groups. Descriptive statistics was calculated depending on the nature of the variables used. Mean ± SD with 95% confidence interval (CI) of the continuous measurements and percentage (%) with 95% CI for categorical measurements were calculated. Bi-variate analysis using simple logistic regression was used to examine the crude relationships between the outcome variables and other independent variables. Considering the cross-sectional design of the study, multiple logistic regression was used to observe any independent relationship between all three forms of undernutrition and elevated BLL while regressing maternal education, maternal BMI, food security status, and household income and gender as potential confounders. Child’s weight (only for stunting), age (only for wasting), length (only for underweight) were also regressed as confounders but not weight, age and length all in the same model in order to avoid multi collinearity. As there is no safe cut-off for BLL, quartiles of BLL was done and regressed in a separate multiple logistic regression model to observe the differential independent relationship of different quartiles of BLL with stunting. Variables in bi-variate analysis with p-value less than 0.15 were added in the multivariate models, as it is unlikely for variables with p-value 0.15 or over to make considerable contribution to multivariate models [38]. Gender of the child has been adjusted in all models as unmodifiable risk factor. All statistical analyses were conducted using STATA 13 (StataCorp. USA). Interactions were not assessed, as the covariates were only used to observe the independent and main effect of elevated BLL on the three forms of undernutrition. Statistical significance was recognized in the multiple logistic regression models, if two-tailed p-value was 0.05 or less and the effect size in the regression model was estimated using odds ratios (ORs) with 95% CI.

## Results

The distribution of the overall characteristics of the sample is shown in Table 2. Of the 729 enrolled subjects whose data was analyzed, over one-third (38.9%) of the children were stunted, around one in every five (19.2%) were wasted (WHZ<-2), around half (51.7%) were underweight (WAZ<-2) and 86.6% children had elevated BLL. Additionally, around half (50.3%) of the children were male, 21.0% of mothers of all children never attended school and 11.1% children were from food insecure households.

**Table 2:** Characteristics of the sample (N = 729).

		n (%)	95% CI
Stunting			
	stunted (LAZ<-2)	284 (39.0)	35.5, 42.5
	not stunted (LAZ≥-2)	445 (61.0)	57.4, 64.5
Wasting			
	wasted (WHZ<-2)	140 (19.2)	16.5, 22.2
	not wasted (WHZ≥-2)	589 (80.8)	77.8, 83.5
Underweight			
	underweight (WAZ<-2)	377 (51.7)	48.1, 55.3
	not underweight (WAZ≥-2)	352 (49.3)	44.7, 51.9
Blood lead level			
	elevated (BLL≥5μg/dL)	631 (86.6)	83.9,88.9
	normal (BLL≤5μg/dL)	98 (13.4)	11.2,16.1
Gender of child			
	Male	367 (50.3)	46.7, 54.0
	Female	362 (49.7)	46.0, 53.3
Maternal Education			
	never attended school	153 (21.0)	18.2, 24.1
	attended school	567 (79.0)	75.9, 81.8
Food Security Status			
	food secure	430 (59.0)	55.4, 62.5
	mildly food insecure	75 (10.3)	8.3, 12.7
	moderately food insecure	143 (19.6)	16.9, 22.7
	severely food insecure	81 (11.1)	9.0, 13.6
		Mean (±SD)	95% CI
HAZ		-1.68 (±1.21)	-1.76, -1.59
WHZ		-0.94 (±1.17)	-1.03, -0.86
WAZ		-1.60 (±1.28)	-1.69, -1.5
BLL (μg/dL)		8.25 (±3.64)	7.98, 8.51
Child’s age (months)		12.6 (±5.18)	12.27, 13.02
Child’s length (cm)		70.8 (±5.67)	70.43, 71.25
Child’s weight (Kg)		7.83 (±1.39)	7.73, 7.93
Maternal height (cm)		149.2 (±6.0)	148.8, 149.7
Maternal weight (Kg)		46.9 (±8.99)	46.4, 47.5
Maternal BMI (Kg/m^2^)		21.0 (±3.6)	20.8, 21.3
Monthly household income (US$)*		124 (±143)	113.5, 134.2

*1 USD = 78 Bangladeshi *Taka*

Table 3 shows the proportions and mean values of characteristics when stratified by stunting categories. The results indicate that significantly higher proportion of stunted children were wasted (p<0.001) and underweight (p<0.001). However, proportion of children with high BLL (p>0.1) and being male (p>0.05) was not significantly different between the not stunted and stunted groups. Significantly higher proportion of stunted children had mothers with no schooling (p<0.05) and belonged to food insecure households (p<0.01).

**Table 3:** Proportions and mean values of characteristics, stratified by stunting categories (N = 729).

		Not stunted, n(%)	Stunted, n(%)	p-value
Wasting				
	Wasted	63 (14.2%)	77 (27.1%)	<0·001
Underweight				
	Underweight	106 (23.8%)	271 (95.4%)	<0·001
Blood lead level				
	elevated (BLL≥5μg/dL)	379 (85.2%)	252 (88.3%)	0.169
Gender of child				
	Male	212 (47.6%)	155 (54.6%)	0.068
Maternal Education				
	never attended school	80 (18.0%)	73 (25.7%)	0.012
Food Security Status				0.001
	Food secure	278 (62.5%)	152 (53.5%)	
	Mildly food insecure	50 (11.2%)	25 (8.80%)	
	moderately food insecure	84 (18·9%)	59 (20.8%)	
	severely food insecure	33 (7.42%)	48 (16·9%)	
		Not stunted, mean (±SD)	Stunted, mean (±SD)	p-value
BLL (μg/dL)		8.10 (±3.80)	8.47 (±3.37)	0.179
Child’s age (months)		11.7 (±4.94)	14.1 (±5.20)	<0·001
Child’s length (cm)		71.7 (±5.65)	69.5 (±5.42)	<0·001
Child’s weight (Kg)		8.28 (±1.34)	7.13 (±1.16)	<0·001
Maternal height (cm)		150.2 (±5.35)	147.7 (±6.62)	<0·001
Maternal BMI (Kg/m^2^)		21.6 (±3.55)	20.1 (±3.40)	<0·001
Monthly household income (USD)*		140 (±175)	99 (±61)	<0·001

*1 USD = 78 Bangladeshi *Taka*

Additionally, there was no significant difference between the mean BLL (p>0.1) among the not stunted and stunted children. However, children who were not stunted had significantly increased mean length (<0·001), weight (<0·001), maternal height (<0·001), maternal BMI (<0·001) and monthly household income (<0·001) comparing to the stunted children. Mean age of the stunted children was significantly higher (<0·001) than the children who were not stunted.

Table 4 shows the relationship between the three forms of undernutrition and elevated BLL among the sampled children. Bi-variate results suggest that elevated BLL was not significantly associated with stunting [OR: 1.37 (95% CI: 0.87–2.15); p>0.05], wasting [OR: 1.37 (95% CI: 0.87–2.15); p>0.05] and being underweight [OR: 1.44 (95% CI: 0.94–2.21); p>0.05]. However, when adjusted for child’s gender, weight, maternal education, BMI, average household income and HFIAS categories, the multiple logistic regression models suggest that elevated BLL was a statistically significant and independent predictor of stunting. The odds of becoming stunted if blood lead concentration was elevated was around twice [aOR: 1.78 (95% CI: 1·06–2.99); p<0.05] in comparison to the odds for those with normal BLL. Additionally, elevated BLL was independently and significantly associated with being underweight [aOR: 1.67 (95% CI: 1·03–2.70); p<0.05] but not with wasting [aOR: 1.18 (95% CI: 0.64–2.19); p>0.05].

**Table 4:** Relationship between the three forms of undernutrition and elevated BLL (N = 729).

Relationship between stunting and elevated BLL*							
		Crude OR	95% CI	p-value	Adjusted OR	95% CI	p-value
	elevated	1.37	0.87–2.15	0.170	1.78	1.07–2.99	0.028
Relationship between wasting and elevated BLL**							
	elevated	1.37	0.76–2.45	0.294	1.18	0.64–2.19	0.581
Relationship between underweight and elevated BLL***							
	elevated	1.44	0.94–2.21	0.096	1.63	1.02–2.61	0.043

*adjusted for child’s gender, weight, maternal education, BMI, average household income and HFIAS categories

**adjusted for child’s gender, age, maternal education, BMI, average household income and HFIAS categories

***adjusted for child’s gender, length, maternal education, BMI, average household income and HFIAS categories

The relationship of other selected variables with stunting, wasting and being underweight along with their effect size is shown in Table 5. Being male [aOR: 2.25 (95% CI: 1.57–3.23); p<0.001] and severely food insecurity household status [aOR: 1.91 (95% CI: 1·07–3·40); p<0.05] increased the odds of being stunted. While, increase in weight [aOR: 0.46 (95% CI: 0.39–0.54); p<0.001] and increased maternal BMI [aOR: 0.94 (95% CI: 0.90–0.99); p<0.05] had protective effect against stunting.

**Table 5:** Other predictors of stunting, wasting and being underweight.

**Other predictors of Stunting**								
		Crude OR	95% CI	p-value	Adjusted OR		95% CI	p-value
Gender of the child								
	Female	Reference			Reference			
	Male	1.32	0.98–1.78	0.068	2.25	1.57–3.23		<0·001
Child’s weight (Kg)^1^		0.48	0.41–0.56	<0.001	0.46	0.39–0.54		<0·001
Maternal education								
	attended school	Reference			Reference			
	never attended school	1.58	1.10–2.26	0.013	1.03	0.67–1.58		0.889
Maternal BMI (Kg/m2)^1^		0.88	0.84–0.92	<0.001	0.95	0.90–0.99		0.048
Average household income **(USD)**^1^		0.99	0.99, 0.99	<0.001	0.99	0.99, 0.99		0.050
HFIAS categories								
	food secure	Reference			Reference			
	mildly food insecure	0.91	0.54–1.54	0.736	0.67	0.38–1.21		0.186
	moderately food insecure	1.28	0.87–1.89	0.205	0.97	0.63–1.52		0.910
	severely food insecure	2.66	1.64–4.32	<0·001	1.91	1.07–3.41		0.028
**Other predictors of Wasting**								
Gender of the child								
	Female	Reference			Reference			
	Male	1.13	0.78–1.64	0.508	1.08	0.73–1.58		0.581
Child’s age **(months)**^1^		1.03	0.99–1.07	0.072	1.03	0.99–1.07		0.089
Maternal education								
	attended school	Reference			Reference			
	never attended school	1.93	1.27–2.92	0.002	1.78	1.14–2.80		0.011
Maternal BMI (Kg/m^2^)^1^		0.85	0.80–0.90	<0.001	0.86	0.80–0.91		<0·001
Average household income (USD)^1^		0.99	0.99, 0.99	<0.001	0.99	0.99, 1.00		0.057
HFIAS categories								
	food secure	Reference			Reference			
	mildly food insecure	2.13	1.22–3.73	0.008	1.80	1.00–3.24		0.050
	moderately food insecure	1.14	0.70–1.88	0.598	0.91	0.54–1.53		0.724
	severely food insecure	1.92	1.10–3.33	0.021	1.16	0.63–2.12		0.631
**Other predictors of being Underweight**								
Gender of the child								
	Female	Reference			Reference			
	Male	1.07	0.80–1.43	0.635	1.15	0.83–1.58		0.392
Child’s length (cm)^1^		0.94	0.92–0.97	<0.001	0.94	0.92–0.97		<0·001
Maternal education								
	attended school	Reference			Reference			
	never attended school	1.59	1.11–2.30	0.012	1.35	0.90–2.02		0.147
Maternal BMI (Kg/m^2^)^1^		0.84	0.80–0.88	<0.001	0.85	0.81–0.89		<0·001
Average household income (USD)^1^		0.99	0.99, 0.99	<0.001	0.99	0.99, 0.99		<0·001
HFIAS categories								
	food secure	Reference			Reference			
	mildly food insecure	1.25	0.77–2.05	0.366	0.93	0.55–1.58		0.801
	moderately food insecure	1.28	0.88–1.87	0.201	0.95	0.63–1.44		0.812
	severely food insecure	2.32	1.40–3.84	0.001	1.35	0.77–2.36		0.289

^1^Continious variable.

As for the other independent predictors of wasting and being underweight derived from the adjusted multiple regression, maternal education status of never attending school [aOR: 1.78 (95% CI: 1·14–2.80); p<0.05] and maternal BMI [aOR: 0.86 (95% CI: 0.80–0.91); p<0.001] were significantly associated with child wasting. Whereas, child’s length [aOR: 0.94 (95% CI: 0.92–0.97); p<0.001], maternal BMI [aOR: 0.85 (95% CI: 0.81–0.89); p<0.001] and average household income [aOR: 0.99 (95% CI: 0.99–0.99); p<0.001] was significantly associated with being underweight.

The result of the multiple logistic regression model where BLL was categorized into quartiles, showed that, after adjusting for child’s gender, weight, maternal education, BMI, average household income and HFIAS categories, odds of being stunted was around thrice for children belonging to the last BLL quartile (Q4) [aOR: 2.78 (95% CI: 1.69–4.60); p<0.001], when compared to the first quartile (Q1) and around two and half times when compared to the second quartile (Q2) [aOR: 2.45 (95% CI: 1.50–4.01); p<0.001] and around one and half times when compared to the third quartile (Q3) [aOR: 1.43 (95% CI: 0.89–2.31); p>0.05] though the relationship was not statistically significant (Table not shown).

## Discussion

This study has found BLL to be a statistically significant independent predictor of the occurrence of stunting when adjusted. The study did not find any significant crude relationship between BLL and stunting but statistical significance was established when adjusted for other variables, indicating the possibility of other variables adjusted to be confounders. The results also indicate that elevated BLL is not a significant predictor of wasting but significantly predicts the occurrence of being underweight. Additionally, odds of stunting was found to increase with increased level of BLL. This finding might be described by the phenomenon where lead builds up slowly in a child due to constant exposure to toxic slum environment, thus causing the chronic condition of stunting, but not causing wasting—the more acute manifestations of undernutrition. Moreover, retrospectively, a stunted child would have reduced organ growth which might be due the detrimental effect of lead poisoning. Lead exposure also suppresses bone marrow activities and causes anemia and ultimately undernutrition [39]. The common tendency of children to chew on or swallow non-food items contributes to the ingestion of lead from contaminated environment [3]. *Pica*, which was shown to have a prevalence of 5.5% among study children in rural Bangladesh [40] could be identified as a major cause of lead ingestion in a slum environment. The association of stunting with poor socioeconomic status as reflected by the household income and food security status is expected based on cited literature [41]. Over 85% of children in this study had elevated BLL, compared to 54% of children in a 2012 study of 919 Bangladeshi children less than 16 years of age. The 2012 study included children aged 5–9, who were shown to have higher BLL than children of other age groups [17]. Therefore, as our sample population only included children up to two years of age we might have drawn a more robust inference about relationship between blood level and stunting if our sample population included children above two years of age.

The elevated BLL observed in subjects of this study could likely have been caused by the use of lead-containing indigenous medicines or by industrial contamination [17]. While leaded gasoline has been banned in Bangladesh since 1999, recent studies have found considerable concentrations of lead in Bangladeshi gasoline, and industries such as battery-making can cause lead contamination of the air and soil [42, 43]. Moreover, lead, yet to be banned in paint, is still prevalent in Bangladesh. A national report suggests that the average concentration of lead in paints used in Bangladesh is 132 times higher than the recommended limit [44] and could be a critical source of lead in the slum environment.

Few studies have previously analyzed the association between undernutrition and BLL. A study in Bangladesh found high BLL to be independently associated with low BMI, but this association was no longer significant after controlling for other factors, indicating that socioeconomic factors could be an important confounder [17]. However, finding from this study shows an association between elevated blood lead level and stunting, even after controlling for maternal and socioeconomic factors.

A study in rats indicated that in animals fed a low-calcium diet, lead exposure resulted in a decline in rate of weight gain and food consumption [45]. The data above support the association between lead exposure and stunting, which could be related to the literature showing an inverse relationship between lead exposure and food consumption, eventually contributing to stunting. Increased food insecurity access has also previously been linked to increased risk for stunting [46] and is in line with our finding.

Several studies have shown an association between increase in BLL and decrease in stature. For each 10μg/dL increase in BLL, a reduction in height of about 1–3 cm has been seen [22, 26, 27, 47–50]. A study in Poland proposed a biological basis for the relationship between BLL and height, suggesting that lead in the blood could interrupt matrix calcification, resulting in stunted long bone growth [51]. In addition to the numerous studies suggesting a relationship between elevated BLL and decreased stature, a study of children and adolescents in the Peruvian Amazon showed that children with elevated BLL had increased risk for stunting [39]. A study of two-year old Mexican children also showed that BLL was inversely associated with length-for-age z-score, and that this association was exacerbated by zinc deficiency [25]. This literature supports the findings above by suggesting that increased BLL could cause decreased height, contributing to a higher likelihood of stunting in children with elevated BLL.

Literature shows that preterm lead exposure has also been associated with preterm delivery and reduced birth weight, both of which have been shown to increase risk of stunting [52–54]. This literature suggests that maternal lead exposure during pregnancy could be an important factor in the relationship between lead poisoning and stunting, but maternal lead status was not included in this study. Maternal height can also be an important predictor of stunting in the child [18, 55]. The above results indicate that the association between a child’s BLL and stunting hold even after adjusting for this important maternal anthropometric risk factor.

However, our result indicates no significant relationship between elevated BLL and the more acute form of undernutrition: wasting. This finding suggests that elevated BLL due to chronic exposure is responsible for the more chronic manifestation of undernutrition–stunting.

Lead exposure is also known to cause impaired immune function, especially in infants and children [12, 13]. As irregular microbial infestation of the gut, known as environmental enteropathy (EE), is associated with stunting, immune damage caused by elevated BLL could be contributing to the cycle of microbial infection, diarrhea, and undernutrition seen in children living in unhygienic conditions [56].

While the relationship between lead poisoning and cognitive damage is well known, the association between elevated BLL and stunting inferred in this paper would justify further exploration on the topic. As 86.6% of the children in this study showed elevated BLL, lead poisoning remains an important issue in Bangladesh. Cost-benefit analysis estimates that for every US$1 spent on reducing lead hazards, there is a benefit of US$17–220, making lead reduction one of the most cost-effective public health practices available [10]. Bangladesh has banned inclusion of lead in gasoline, a significant source of lead poisoning in 1999 but Bangladesh does not have any lead screening program for children [17] nor have any guidelines for using lead based paints in households. The data presented above strengthen the argument for improved lead reduction practices in Bangladesh which would produce a significant impact on the Government’s plan to improve children’s nutrition status in Bangladesh.

## Conclusion

This study showed statistically significant association between elevated BLL and stunting in young children. This link between BLL and stunting adds to the existing body of evidence demonstrating the negative effects of lead on the cognitive and immune systems in children, and further illustrates that countries suffering from severe undernutrition should not overlook lead control in their efforts to improve the physical and mental growth and development of their children.

## Strengths and limitations

All study subjects live in the same urban slum area, minimizing possible differences in lead exposure among subjects. This study did not measure environmental lead exposure or proximity to sources of lead contamination, only blood lead level, and thus cannot fully account for differences in lead exposure in the children. Additionally, as it is a cross-sectional study, direction of the effect cannot be established, hence it is also possible that stunting may significantly cause accumulation of lead in blood. Moreover, there are likely to be more factors such as dietary intake which was unadjusted for in the final regression models. This study is not generalizable to represent all Bangladeshi children as data was collected only from slum children with low socio-economic status and who were also more prone to experience pollution.

## Supporting information

S1 Dataset Data_Set. (XLSX)
